# Numerical analysis and experimental verification of optical scattering from microplastics

**DOI:** 10.1098/rsos.230586

**Published:** 2023-08-09

**Authors:** Sinan Genc, Kutay Icoz, Talha Erdem

**Affiliations:** Department of Electrical-Electronics Engineering, Faculty of Engineering, Abdullah Gül University, Kayseri 38080, Turkey

**Keywords:** optical scattering, Mie theory, microparticles, microplastics

## Abstract

Accurate and fast characterization of the micron-sized plastic particles in aqueous media requires an in-depth understanding of light interaction with these particles. Due to the complexity of Mie scattering theory, the features of the scattered light have rarely been related to the physical properties of these tiny objects. To address this problem, we reveal the relation of the wavelength-dependent optical scattering patterns with the size and refractive index of the particles by numerically studying the angular scattering features. We subsequently present a low-cost setup to measure the optical scattering of the particles. Theoretical investigation shows that the angular distribution of the scattered light by microplastics carries distinct signatures of the particle size and the refractive index. The results can be used to develop a portable, low-cost setup to detect microplastics in water.

## 1. Introduction

Within 50 years following the first reports on microplastic pollution in water resources and oceans, microplastics have become a global problem [1–3]. In the last decade, the number of microplastics in oceans and water resources has reached levels that cannot be underestimated. Once these pollutants are released into the seas or clean water sources, they eventually integrate with the marine food chain and affect the ecosystem [4,5]. Detecting these microparticles in commercial seafood proved that human exposure to microplastics via the food chain and water consumption threatens human health [6–9].

The primary sources of microplastic particles are chemical wastes, including toothpaste, detergents, cosmetics, clothes, etc. and the secondary sources of microplastics are mechanical degradation of bigger plastics over time [10–14]. In addition, the existence of microplastics has been proven in different environments, i.e. air, human blood, baby diapers, table salt, honey and sugar [15–23]. Air also carries microplastics as a part of the water cycle. Evaporated water moves pollutants to the air, and via breathing the human body is affected by those particles. As an alternative, rains bring back those pollutants to the ground. So, once those pollutants accumulate in the resources, detecting and eliminating them is difficult. Therefore, classification and definition of the origin of those microplastics are crucial to take precautions at zero-point. The cost-effective detection of microparticle pollution in the water can enable on-site analysis before any hazardous consumption [24]. The classification of microplastics in liquid media is a challenging task due to the variations in shape, size, concentration, and distribution of the particles.

Regarding the theoretical analysis of a sample with only one type of material, multiple scattering should be considered due to the enormous number of particles. The analytical solution to this problem is highly complex. Scattering theory is one of the most common methods to characterize particles in aqueous media [25].

Mie scattering theory, named after German physicist Gustav Mie, is a fundamental concept in optics that describes the interaction between electromagnetic radiation and spherical particles. It provides a mathematical framework for comprehending the scattering of light or other electromagnetic radiation by particles of comparable size to the incident radiation's wavelength. Mie scattering theory considers both the incident wave and the scattered wave as a series of spherical wave components, and it accounts for various parameters such as particle size, refractive index, and polarization of the incident light [26–30].

Although Mie's theory provides scattering cross-sections in terms of angular intensity functions of a single homogeneous sphere, the calculations get complicated when the number of scatterers increases, even if they are identical.

The plastic particles having a size between 1 µm and 5 mm are termed microplastic, whereas particles less than 1 µm are called nano plastics. Many studies report the toxicity of microplastics and nano plastics, emphasizing a demand for a consistent and reliable methodology to detect and characterize those pollutants [31–33]. At high concentrations of microparticles, the effect of multiple scattering from numerous particles would be unavoidable. At lower concentrations, however, the optical scattering may be approximated to single-particle scattering allowing for theoretical analysis. With this motivation, here, we aim to obtain a low-cost measurement setup to record the optical scattering patterns of microparticles in water. We used the Mie theory for a single particle and single scattering event to estimate the optical response of the particles by focusing on the particles having a size of 1–10 µm. The acquired scattering images are subsequently processed to obtain angle-dependent relative scattering intensity, where the effect of the particle size and refractive index on the optical scattering patterns are analysed and compared. This low-cost setup and the image processing tool can be further optimized into a portable system to detect microplastics in water.

## 2. Theory

Mie scattering theory enables the prediction and interpretation of scattering patterns, such as the intensity, phase, and angular distribution of the scattered radiation, by solving Maxwell's equations for electromagnetic waves interacting with spherical particles. This theory has applications in numerous disciplines including atmospheric physics, aerosol science, remote sensing, and nanoparticle research [34–36]. To analyze the scattering from micro-sized particles, we employed the Mie scattering cross-sections. The derivation of these cross-sections obtained using the equations given in [27–30] was presented in electronic supplementary material.

In Mie theory, differential scattering cross-sections ($\sigma_{\mathrm{VV}}^{\prime }$, and $\sigma_{\mathrm{HH}}^{\prime }$) are defined as given in equations (2.1) and (2.2)

2.1 $$\sigma_{\mathrm{VV}}^{\prime }=\frac{\lambda^{2}}{4\pi^{2}}|S_{1}(\theta ){|}^{2},$$

and

2.2 $$\sigma_{\mathrm{HH}}^{\prime }=\frac{\lambda^{2}}{4\pi^{2}}|S_{2}(\theta ){|}^{2},$$

where λ is the wavelength of incident light beam and the subscript VV refers to vertically polarized incident light and vertically polarized scattered light with respect to the incident scattering plane. Similarly, HH refers to horizontally polarized incident light and horizontally polarized scattered light. The remaining pairs, VH and HV, have extremely insignificant contributions which are ignored in general [29,37,38]. Scattering intensity functions, $S_{1}\;\mathrm{and}\;S_{2}$ are defined by vertically and horizontally polarized incident light which are given in equations (2.3) and (2.4), respectively.

2.3 $$S_{1}(\theta )=\overset{\infty }{\underset{n=1}{\sum }}\frac{2n+1}{n(n+1)}[a_{n}\pi_{n}(\cos\theta )+b_{n}\tau_{n}(\cos\theta )],$$

and

2.4 $$S_{2}(\theta )=\overset{\infty }{\underset{n=1}{\sum }}\frac{2n+1}{n(n+1)}[a_{n}\tau_{n}(\cos\theta )+b_{n}\pi_{n}(\cos\theta )],$$

where $\pi_{n}$ and $\tau_{n}$ express Legendre polynomials as given in equations (2.5) and (2.6), respectively.

2.5 $$\pi_{n}(\cos\theta )=\frac{P_{n}^{\left(1\right)}(\cos\theta )}{\sin\theta },$$

and

2.6 $$\tau_{n}(\cos\theta )=\frac{dP_{n}^{\left(1\right)}(\cos\theta )}{d\theta },$$

where parameters $a_{n}$ and $b_{n}$ are defined as given in equations (2.7) and (2.8), respectively.

2.7 $$a_{n}=\frac{n_{\mathrm{med}}{\Psi^{\prime }}_{n}(n_{\mathrm{part}}x)\Psi_{n}(x)-n_{\mathrm{part}}\Psi_{n}(n_{\mathrm{part}}x){\Psi^{\prime }}_{n}(x)}{n_{\mathrm{med}}{\Psi^{\prime }}_{n}(n_{\mathrm{part}}x)\zeta_{n}(x)-n_{\mathrm{part}}\Psi_{n}(n_{\mathrm{part}}x){\zeta^{\prime }}_{n}(x)},$$

and

2.8 $$b_{n}=\frac{n_{\mathrm{part}}\Psi_{n}^{\prime }(n_{\mathrm{part}}x)\Psi_{n}(x)-n_{\mathrm{med}}\Psi_{n}(n_{\mathrm{part}}x)\Psi_{n}^{\prime }(x)}{n_{\mathrm{part}}\Psi_{n}^{\prime }(n_{\mathrm{part}}x)\zeta_{n}(x)-n_{\mathrm{med}}\Psi_{n}(n_{\mathrm{part}}x)\zeta_{n}^{\prime }(x)},$$

where $n_{\mathrm{part}}$ is the refractive index of the particle, $n_{\mathrm{med}}$ is the refractive index of the medium, and size parameter, *x*, is defined by equation (2.9),

2.9 $$x=\frac{2\pi an_{\mathrm{med}}}{\lambda },$$

where λ is the wavelength of the incident light. The Ricatti-Bessel functions, Ψ, and ζ are defined by the half-integer-order Bessel function of the first kind as in equations (2.10) and (2.11), respectively, where

2.10 $$\Psi_{n}(z)={\left(\frac{\pi z}{2}\right)}^{1/2}J_{n+1/2}(z),$$

and

2.11 $$\zeta_{n}(z)={\left(\frac{\pi z}{2}\right)}^{1/2}H_{n+1/2}(z)=\Psi_{n}(z)+iX_{n}(z).$$

$H_{n+1/2}(z)$ is the half-integer-order Hankel function of the second kind and $X_{n}(z)$ is defined by half-integer-order Bessel function of second kind $\left(N_{n+1/2}(z)\right)$ as presented in equation (2.12),

2.12 $$X_{n}(z)\;=\;-{\left(\frac{\pi z}{2}\right)}^{1/2}N_{n+1/2}(z).$$

Using the equations given above, the differential scattering cross-sections ($\sigma_{\mathrm{VV}}^{\prime }$ and $\sigma_{\mathrm{HH}}^{\prime }$) are calculated and their average represents the randomly polarized (unpolarized, $\sigma_{\mathrm{scat}}^{\prime }$) incident light differential cross-section. We identified the angles of the first four scattering peaks using MATLAB's built-in function to find peaks theoretically.

## 3. Numerical method

In literature, small particles are approximated to spheres to make analytical solutions possible and decrease the computational cost of numerical solutions. It is already complicated to solve scattering equations in multiple scattering regimes. If features such as surface roughness and the shape of the particles come into play, it would be challenging and costly to obtain an exact solution. Thus, in this study, as is common in the literature, we did calculations and simulations for spherical particles in a single particle regime [39,40]. Although it sounds inappropriate to use single scattering equations for a case involving many particles, as the study considers interference ring angles, the match between theory and experiments shows that it is possible to use the same equations. The details and results will be explained in the following parts.

For numerical calculations, to avoid complexity of the Ricatti-Bessel functions $\Psi_{n}$ and $\zeta_{n}$, the Lorenz-Mie coefficients (equations (2.7) and (2.8)) are rewritten in a form containing only their ratios between them as given in equations (3.1) and (3.2) [41].

3.1 $$a_{n}=\frac{\Psi_{n}(x)}{\zeta_{n}(x)}\frac{n_{\mathrm{med}}A_{n}(n_{\mathrm{part}}x)-n_{\mathrm{part}}A_{n}(x)}{n_{\mathrm{med}}A_{n}(n_{\mathrm{part}}x)-n_{\mathrm{part}}B_{n}(x)},$$

and

3.2 $$b_{n}=\frac{\Psi_{n}(x)}{\zeta_{n}(x)}\frac{n_{\mathrm{part}}A_{n}(n_{\mathrm{part}}x)-n_{\mathrm{med}}A_{n}(x)}{n_{\mathrm{part}}A_{n}(n_{\mathrm{part}}x)-n_{\mathrm{med}}B_{n}(x)}.$$

Here, $A_{n}$ and $B_{n}$ are logarithmic derivatives of $\Psi_{n}(z)$ and $\zeta_{n}(z)$ as presented in equations (3.3) and (3.4), respectively:

3.3 $$A_{n}=\Psi_{n}^{\prime }(z)/\Psi_{n}(z),$$

and

3.4 $$B_{n}=\zeta_{n}^{\prime }(z)/\zeta_{n}(z).$$

The ratio $A_{n}$ is only numerically stable with downward recurrence. Therefore, equation (3.5) is employed for its evaluation [41].

3.5 $$A_{n}(z)=\frac{n+1}{z\;}-\;{\left(\frac{n+1}{z}+A_{n+1}(z)\right)}^{-1}.$$

Even though equation (3.5) is valid for the ratio $B_{n}$, it is regrettably unstable for both upward and downward recurrences [42]. For $B_{n},$ a different formula has been devised [43] in the field of multi-layered particles embedded in a non-absorbing medium. Any complex argument is numerically stable with upward recurrence [43]:

3.6 $$B_{n}(z)=A_{n}(z)+\frac{i}{\Psi_{n}(z)\zeta_{n}(z)},$$

and

3.7 $$\Psi_{n}(z)\zeta_{n}(z)=\Psi_{n-1}(z)\zeta_{n-1}(z)\left(\frac{n}{z}-A_{n-1}(z)\right)\left(\frac{n}{z}-B_{n-1}(z)\right).$$

Equations (3.1) and (3.2) require a recurrence relation for the ratio $\Psi_{n}(z)/\zeta_{n}(z)$.

3.8 $$\frac{\Psi_{n}(z)}{\zeta_{n}(z)}=\frac{\Psi_{n-1}(z)}{\zeta_{n-1}(z)}\frac{B_{n}(z)+n/z}{A_{n}(z)+n/z}.$$

The amplitude functions, equations (2.3) and (2.4), are defined by an infinite sum; therefore, in order to obtain a reasonable approximation, an appropriate number of terms, M, must be determined. This is also required to initialize the downward recurrence (equation (3.5)) that computes $A_{n}(x)$ and $A_{n}(n_{\mathrm{part}}x)$. A formula for determining M, supported by empirical [43,44] and theoretical [42] evidence, is

3.9 $$M=[|x|+p{\left|x\right|}^{1/3}],$$

where *p* = 4.3 gives a maximum error of $10^{-8}$. It is possible to calculate an approximate initial value for the downward recurrence (equation (3.5)), but, as explained in [45], the recurrence is not sensitive to the initial value, and therefore it can be arbitrarily chosen as $A_{M}(z)=0.$

Once $A_{0}(z),\ldots \;,A_{M}(z)$ have been calculated for z=x and z=y, it is possible to find the ratios $B_{n}(x)$ and $\Psi_{n}(x)/\zeta_{n}(x)$ as well as $a_{n}$ and $b_{n}$. There is no need to store $B_{n}(x)$ and $\Psi_{n}(z)/\zeta_{n}(z)$ since they are computed using upward recurrences (equations (3.6)–(3.8)). These recurrences should be initialized by

3.10 $$B_{0}(z)=i,$$

3.11 $$\Psi_{0}(z)\zeta_{0}(z)=\frac{1}{2}(1-e^{i2z}),$$

3.12 $$\mathrm{and}\;\Psi_{0}(z)/\zeta_{0}(z)=\frac{1}{2}(1-e^{-i2z}).$$

Recall that the wavelength λ and size parameter *x* have a direct relationship. This indicates that the Lorenz-Mie coefficients are wavelength-dependent and should be sampled at various wavelengths. They also depend on the particle radius r, and are applicable to spherical particles of arbitrary size as long as they do not exhibit diffuse reflection (which is only possible if the particle size considerably exceeds the wavelength, although the particle surface may still be smooth [28]). As input parameters for computing the optical properties of a scattering material, the properties of each particle inclusion and the host medium are necessary. This robust method of calculating the Lorenz-Mie coefficients permits the calculation of scattering amplitudes $S_{1}$ and $S_{2}$ (equations (2.3) and (2.4)). These allow for the calculation of extinction and scattering cross-sections as well as the particle's phase function.

Using MATLAB, we embedded all equations into a function to calculate angular scattering distribution by a particle [46]. The inputs of the functions are the diameter of the sphere, wavelength of incident light, and refractive indices of the sphere and the medium. At the output differential scattering cross-section data and first four scattering peak angles were obtained up to 21.8° to match the experimental results. Considering the wavelength of the incident light, we determined the refractive index of the medium, which is that of water in this study.

The function calculates the peak angles of bright rings on the scattering pattern within the determined angular range. These results were used to plot the scattering intensity with respect to angle from 5° to 21.8°. This range of angles depends on the distance of the screen from the sample and the size of the screen. It is possible to have scattering information of wider angles by enlarging the screen and placing it closer to the sample. However, the intensity of scattered light would go lower through the outer angles. Increasing the light power would cause saturation at the centre. Therefore, the results are analysed starting from 5° to remove saturation risks. The size of the screen and settlement of the setup provided the maximum limit of 21.8° and in that region four or five peaks are observed. Since we are able to clearly distinguish individual scattering rings within this angular range, we analysed our experimental and theoretical results in this range.

To account for the refraction event while the light passes from the aqueous medium to air, we employed Snell's law. We calculated the angular distribution of the scattered light in the air. For this calculation, we used the refractive indices of the water at the corresponding wavelength and air in addition to the angular distribution of the scattered light in water.

## 4. Experiment

**Figure 1.**
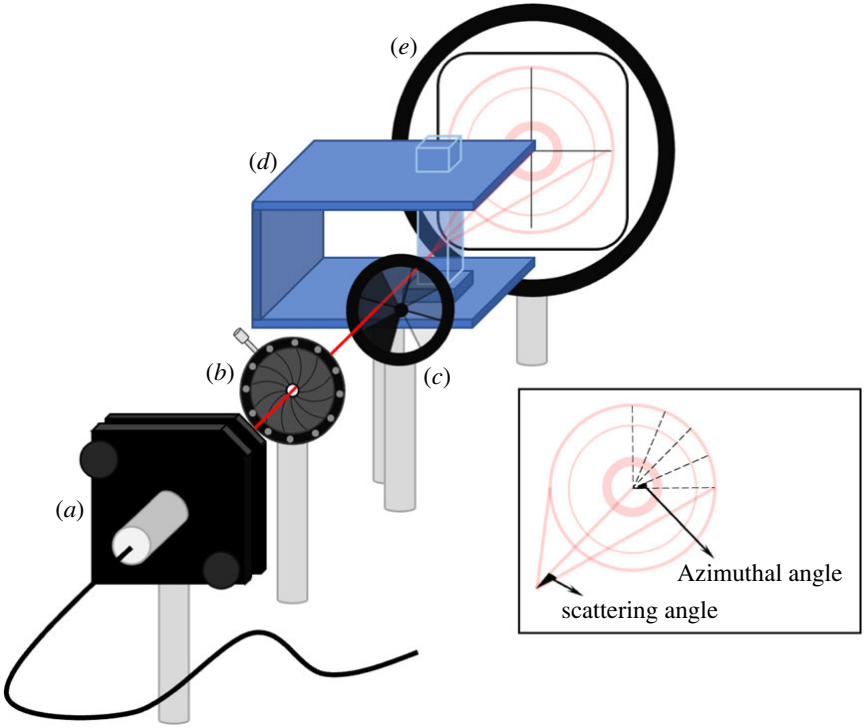
Illustration of the experimental setup, (*a*) light source, (*b*) iris, (*c*) neutral density filter, (*d*) cuvette holder and cuvette, and (e) graded white screen, (inset) azimuthal and scattering angles.

For the measurements, at room temperature (22°C), the laser power was kept at 170 µW for collimated red, green and blue lasers, commercially available CPS405, CPS520 and CPS650F-THORLABS, emitting at 405 nm, 520 nm and 650 nm, respectively. However, their data sheets report more precise values, 403.8 nm, 514.9 nm and 656.3 nm, which we preferred to use. The beam was directed through an iris to clear the beam. Just after the iris, a neutral density filter was used to adjust the laser power to the same level during the experiments. A cuvette holder was three-dimensionally printed to keep the cuvette in the optimum orientation. Finally, a graded white screen was placed to have the scattering pattern on it, as presented in figure 1.

**Figure 2.**
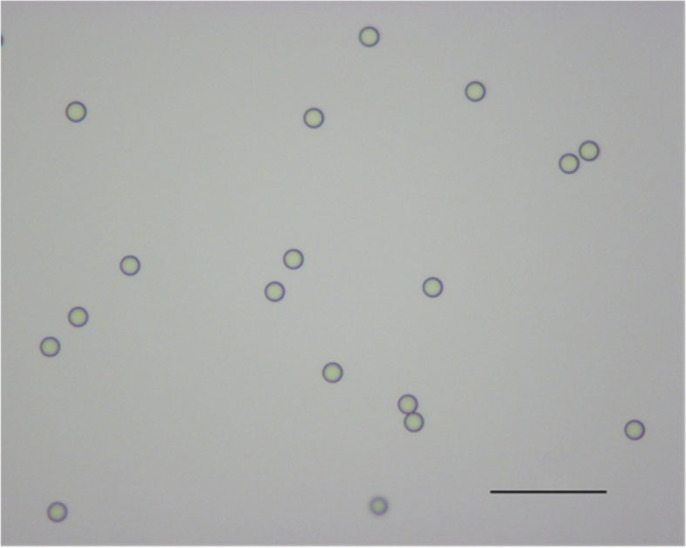
40x zoomed microscope image of melamine particles. Scale bar: 50 µm.

Commercially available 8 µm ± 100 nm-sized melamine resin (Me) (95523-Sigma Aldrich) microspheres were used in the experiments to compare between the predictions of Mie theory that we calculated numerically and the experimental results. The microscope image of melamine spheres is given in figure 2.

Samples were prepared at concentrations from 0.05 fM up to 3.00 fM by adding them into ultra-pure water using a micropipette and kept in vials. The samples were shaken using a vortex before the experiments to make the solution much more homogeneous and by hand just before the measurements one more time. In our experiments, the safe time limit was about 5 min. The sinking of the particles deeper in the cuvette and a more than 5% decrease in scattering intensity were observed after 5 min. Our measurement time for each sample was much shorter than this limit. In addition, the 0.05–3.00 fM range corresponds to 3 × 10^4^–180 × 10^4^ particles ml^−1^, higher than the values studied in the literature. However, the sizes of the particles reported in the literature are about 10–100 times bigger, significantly increasing the scattering cross-section [23]. Considering this size difference, we believe it is reasonable to have a 0.05 fM–3.00 fM concentration range in our experiments for the particle sizes we are interested in.

Subsequently, each sample was placed into cuvettes, and the scattering patterns of the laser light at different wavelengths from these particles fell on a screen placed 6.5 cm apart from the cuvette. 200 images of these scattering patterns were taken in a dark environment by a CMOS camera (Raspberry Pi Focus Adjustable Camera Module-2592 × 1944 pixels), 100 ms shutter speed and 20 ms exposure time, controlled by Raspberry Pi 4–4GB RAM. In total, 1800 images are recorded for processing.

There is an uncertainty in the literature on regarding the refractive index of melamine particles. Thus, we studied the range given in literature which is 1.530–1.922 for the red wavelength range [47–49]. Since melamine has different forms with different refractive indices leading to different refractive indices, comparison of theoretical and experimental results became unclear. We found the most satisfying match with the Mie theory and experiments when the refractive index is 1.79 at 656.3 nm. On the other hand, based on the data in the literature, we used refractive indices of 1.89 and 1.96 for 514.9 nm and 403.8 nm, respectively.

## 5. Image processing

**Figure 3.**
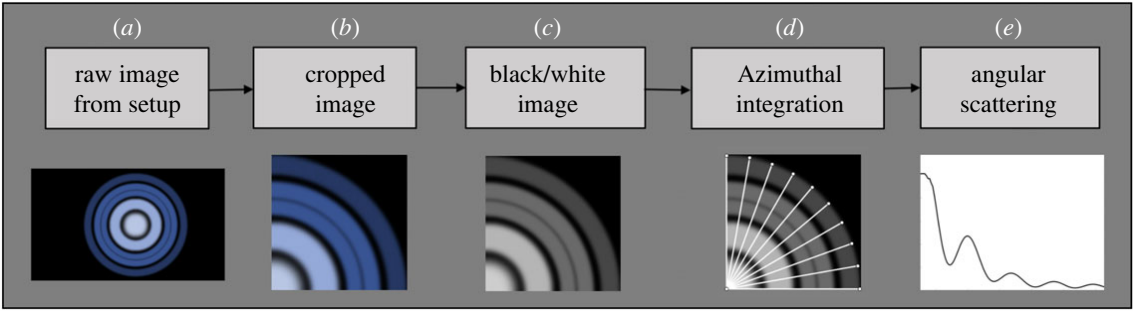
Representation of image processing flow from the raw image to the scattering pattern.

The images of the scattering patterns (figure 3*a*) were then analysed numerically. To minimize noise, the average of 200 images was calculated for each sample's scattering pattern. Next, as illustrated in figure 3*b,c*, all the images were cropped and converted to grayscale. To decrease the computational cost, 86 lines were defined with 1° azimuthal angle increments between 5° and 90° starting from the centre towards the outer regions on the upper-right quartile of the images as given in figure 3*d*, (figure 3*d* presents only 10° azimuthal increment for better visualization). As the next step, the average pixel data on all those 81 lines was taken to decrease the noise level on measurements. Finally, azimuthal angular scattering intensity was obtained, as presented in figure 3*e*. However, it was still noisy to identify peak angles, therefore 2nd-degree polynomial was fitted to the experimental data around each peak because fitting a polynomial with an exact high degree, i.e. 25th, was not giving an appropriate match for every scattering data point. We assumed that fitting a 2nd-degree polynomial will provide consistent methodology during the analysis. Therefore, using an angle range from the left and right side of each peak, we fit a 2nd-degree polynomial and used the angle of the peak point as experimental peak angles.

## 6. Results and discussion

**Figure 4.**
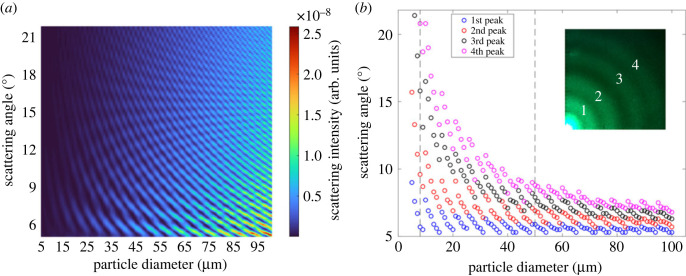
(*a*) Dependence of the scattering intensity on the particle size as calculated using Mie theory. The particle size varied between 5–100 µm in water and 514.9 nm incident light beam in MATLAB; (*b*) angular distribution of the first four scattering intensity peaks to the particle size as calculated using Mie theory. The particle size was varied between 5–100 µm in water and 514.9 nm incident light beam in MATLAB, (inset) experimental image showing peaks (greater than 5°) for 8 µm.

As given in equation (2.9), the size parameter is directly proportional to the particle diameter. In the same medium (water) and using the same wavelength of the incident light, the effect of particle size was investigated to study angular scattering changes based on numerical calculations. In figure 4*a*, the peaks of the scattering angles as a function of particle size were presented for the green light (at a wavelength of 514.9 nm) and water (*n* = 1.3344). The particle diameter increases from 5 to 100 µm with a step size of 1 µm.

Next, the angular scattering patterns were calculated numerically using Mie theory. The gap between the peaks of the scattering angles gets narrower when the particle size increases. Having a bigger particle means that the forward scattering will be more dominant. In a defined range of scattering angles, the number of peaks will increase with the size of the particle because the scattering pattern will get closer to the horizontal axis. For instance, as given in figure 4*b*, for an 8 µm particle, the widest visible peak, which is the fourth peak, is around 21°. However, for a bigger particle, i.e. 50 µm, the fourth peak appears around 9° which shows that when particle size is bigger, the side peaks (around zero degrees) get closer to the zero degrees, revealing that the forward scattering is dominant.

**Figure 5.**
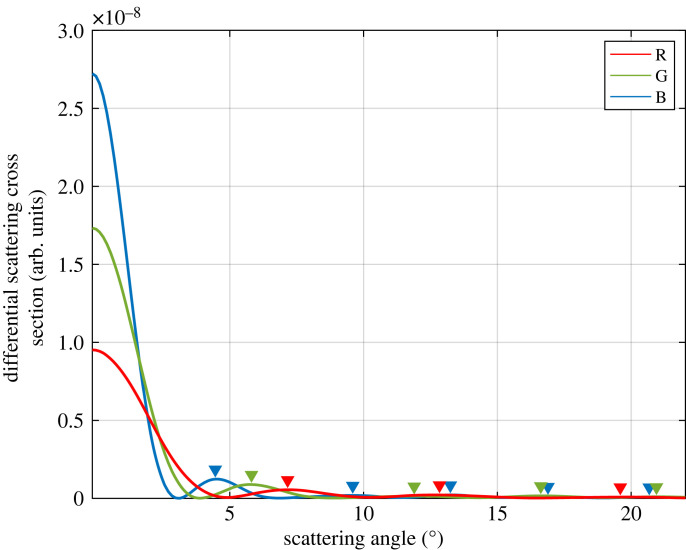
Calculated angular distribution of the scattering cross-section of 8 µm-sized particle in water for three different wavelengths of incident light. The triangles represent the locations of the scattering peaks.

An experimentally acquired image belonging to 8 µm melamine particle for green incident light is given, and the peak angles were demonstrated in figure 4*b* (inset). Subsequently, the effect of the laser wavelength was studied. The refractive index of the medium also was adjusted considering this change. As shown in figure 5 (normalized version in electronic supplementary material, figure S1), there are more peaks at shorter wavelengths than the longer wavelengths. In Mie scattering, the intensity of scattered light depends on both the size and the refractive index of the particle. The behaviour of Mie scattering is more complex and does not follow a simple wavelength dependency like Rayleigh scattering which occurs when size is small compared to the wavelength. In the Mie regime, different wavelengths of light can lead to different scattering patterns, variations in the intensity, and angular distribution of scattered light [50]. For instance, shorter wavelengths (blue or ultraviolet light) are more strongly scattered by small particles, whereas longer wavelengths (red or infrared light) may penetrate deeper into the particle and experience less scattering. Thus, we observe that when the wavelength of the beam shortens, the forward scattering becomes more dominant.

Furthermore, within the same range of angles, we see more peaks by blue light. In other words, the bright circle at the centre gets narrower at blue end of the visible spectral range. Therefore, blue light scatters stronger while the red light scatters weaker. In addition, the first bright ring angle occurs at around 5° for blue light and around 7° for the red light which means the forward scattering forms an intense but squeezed circle for blue light. The same effect becomes visible in the number of peaks within the angular range that we analysed: scattering of the blue light produced five distinct peaks while four and three peaks appeared when green and red lasers were employed, respectively, although all the incident lights have the same optical power (figure 5, normalized version electronic supplementary material, figure S1).

**Figure 6.**
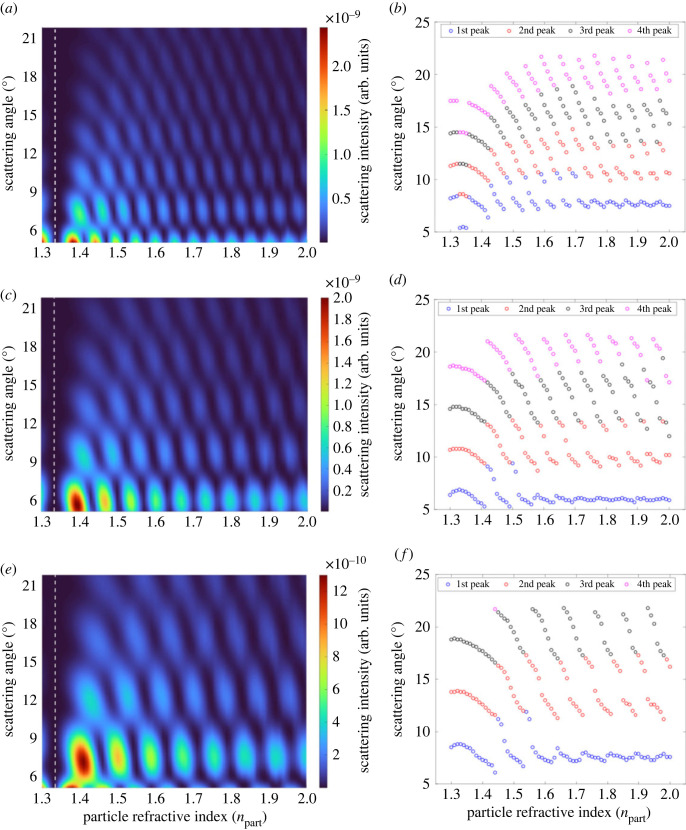
Change of refractive index between 1.3 and 2.0 for Me particles at 8 µm excited by a laser operating at (*a*,*b*) 403.8 nm, (*c*,*d*) 514.9, and (*e*,*f*) 656.3 nm as wavelengths of incident light. The white dashed line indicates the refractive index of water (1.33) where there is no scattering.

In figure 6, the effect of the refractive index is numerically calculated for the same particle size and medium at (a–b) 403.8 nm, (c–d) 514.9 nm and (e–f) 656.3 nm as wavelengths of incident light, respectively. As presented in these figures, when the refractive index $\left(n_{\mathrm{part}}\right)$ increases, the particle scatters the light weaker at lower angles and stronger at larger angles. Furthermore, the angle of each peak occurs at higher values. For instance, in figure 6*a*, we observe the fourth peak around 14–15° for a refractive index of about 1.4, whereas it shows up about 18–20° for a particle with a refractive index of 1.9–2.0.

To compare the results obtained from theory and experiments, we measured three samples at concentrations ranging from 0.05 fM to 3.00 fM. The average angle of each peak was calculated corresponding to the angular scattering of different concentrations for Me particles of 8 µm. The peak angles obtained from the theory are similar to the ones obtained from the average of each experimental data for red, green, and blue wavelength of the incident lights, respectively.

**Figure 7.**
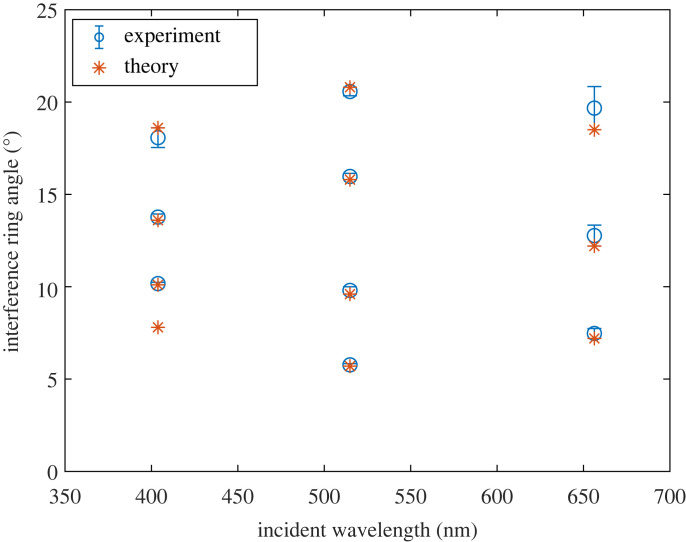
Average and standard deviation of the peak angles recorded in three experiments and the theoretical angles calculated using the Mie theory.

As presented in table 1, the difference between average peak angles measured in the laboratory and calculated theoretically is about one degree. Furthermore, to monitor consistency among measurements, after preparing fresh sets of samples, the same experiments were held at least three months later than the previous one. Standard deviations of the obtained peak angles among three experimental results for red, green, and blue incident lights were calculated separately for each peak. The results are at most 0.1°, 0.1°, and 0.2°, respectively, indicating the consistency between independent measurements. In figure 7, the average and standard deviation of the peak angles recorded in three experiments and the angles obtained using the Mie theory are given.

**Table 1.** Comparison of numerical calculations and an average of three experimental measurements of the first four bright peak angles (P1, P2, P3, P4) of 8 µm-sized Me particles and percentage errors for each peak between those two types of results.

	Mie theory			experiment			STD			Percentage errors (%)		
	656.3 nm	514.9 nm	403.8 nm	656.3 nm	514.9 nm	403.8 nm	656.3 nm	514.9 nm	403.8 nm	656.3 nm	514.9 nm	403.8 nm
**P1** (**°)**	7.2	5.7	7.8	7.5	5.8	NA	0.12	0.12	NA	4.16	1.75	NA
**P2** (**°)**	12.2	9.6	10.1	12.8	9.8	10.1	0.06	0.10	0.06	4.91	2.08	0.00
**P3** (**°)**	18.5	15.8	13.6	19.7	15.9	13.8	0.15	0.06	0.21	6.48	0.63	1.47
**P4** (**°)**	NA	20.8	18.6	NA	20.6	18.1	NA	0.06	0.15	NA	0.96	2.69

The difference between the average of measured peaks and calculated ones is about 1° with the largest deviations occurring when the red laser is employed (figure 7 and table 1). For green, the match between theory and the experiment is the best.

**Figure 8.**
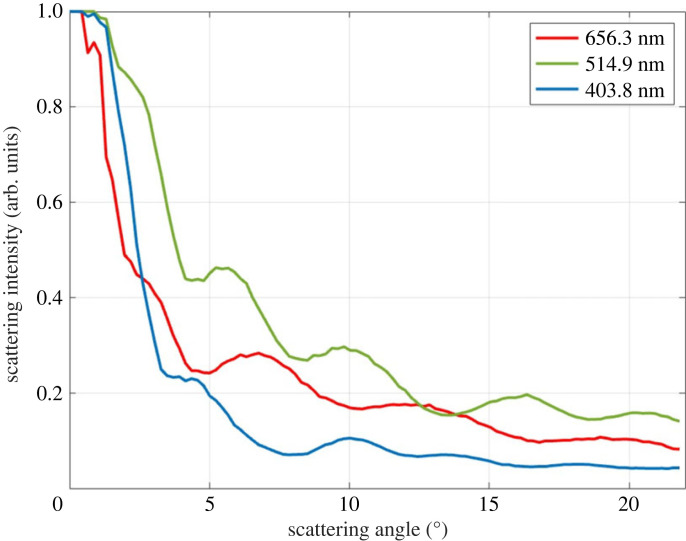
Angular scattering of 1.50 fM melamine particles by red, green and blue lasers.

As presented in figure 8, while blue light provides weaker scattering, it has more peaks compared to others. Considering the simulation results for wavelength of incident light in figure 5, as expected, from blue wavelength range to red wavelength range, the number of peaks decreases in the same range. Different than the theoretical figure 5, the intensity of blue peaks is less than the others. We assume that this happens due to the laser spot and wide saturated spot at the centre. It has an effect on intensity. However, peak angles match with the theoretical values as presented in table 1.

In addition, considering the difference between peak angles for red, green, and blue wavelength of incident lights, we calculated the root mean squared error (RMSE) values and averaged them to find the particle size which has the closest fit to experimental results. By 0.3°, 0.5°, and 0.3° degrees of RMSE values of red, green, and blue beams, respectively, the best-fitted angular spectrum also belongs to an 8-μm particle. The refractive indices for these estimated particles are at most 0.06 different than the original refractive indices for related wavelengths. The percentage errors between theoretical and experimental values of each peak are also given in table 1. As presented, percentage errors wave between 0.00 and 6.48% and have high values for the red incident light. As expected, the same amount of difference, i.e. 1°, causes higher percentage errors in first peak compared to fourth peak.

We anticipate that the resolution of the camera causes this difference due to the limited pixel size and response performance to different colours and the number of pixel intensities collected on each azimuthal line on images. Although averaging multiple images was implemented to decrease the noise, we anticipate there was still some low noise effect on images.

Having a computational method that is based on the particle's size, refractive index, wavelength of the incident light and refractive index of the medium would make all complex calculations easier to get scattering information. Obtaining angular scattering of particles provides a broad window for investigating those particles and their scattering behaviours. A cost-effective setup, providing that small error between experiments and numerical calculations, can be developed and support the research in this field.

## 7. Conclusion

Although larger particles are easier to detect using low-cost methods, further analyses of small particles are highly desired [26]. Keeping this in our focus, here, we uncovered the relations between the scattering pattern and microplastic characteristics in water by utilizing Mie scattering for particles between 1 and 100 µm.

Mie theory calculations show that the scattering patterns carry distinct signatures of the particle size and refractive index that also depend on the wavelength of the incident light. For example, the light intensity increases around 0° with increasing particle size. In addition, the angular distribution of the scattering pattern becomes wider as the particle size increases. Furthermore, increasing the refractive index makes the angular distribution of the scattering pattern denser. We also observed that the scattering pattern becomes wider at longer wavelengths of the incident light.

We employed a low-cost, portable system with three lasers operating in red, green and blue wavelength ranges of the visible spectrum. We analysed melamine as the model material and revealed that the experimental results match the theoretical calculations within 1° variation. This portable low-cost measurement system for microplastics classification by optical scattering patterns can be further optimized to monitor smaller size microplastics and even nano plastics in water.

## Data Availability

MATLAB code used for numerical calculations can be found at https://github.com/sinanngenc/miemtlb. The detailed equations supporting this article have been uploaded as part of the electronic supplementary material [52]. Data and relevant code for this research work are stored in GitHub: https://github.com/sinanngenc/miemtlb and have been archived within the Zenodo repository: https://doi.org/10.5281/zenodo.8134110 [46,51].

## References

[1] Ashkin A. 1970 Acceleration and Trapping of Particles by Radiation Pressure. Phys. Rev. Lett. **24**, 156. (10.1103/PhysRevLett.24.156) 10030975

[2] Carpenter EJ, Anderson SJ, Harvey GR, Miklas HP, Peck BB. 1972 Polystyrene Spherules in Coastal Waters. Science (1979) **178**, 749-750. (10.1126/SCIENCE.178.4062.749) 4628343

[3] Carpenter EJ, Smith KL. 1972 Plastics on the Sargasso Sea Surface. Science (1979) **175**, 1240-1241. (10.1126/SCIENCE.175.4027.1240) 5061243

[4] do Sul JAI, Costa MF. 2014 The present and future of microplastic pollution in the marine environment. Environ. Pollut **185**, 352-364. (10.1016/J.ENVPOL.2013.10.036) 24275078

[5] Wright SL, Thompson RC, Galloway TS. 2013 The physical impacts of microplastics on marine organisms: a review. Environ. Pollut **178**, 483-492. (10.1016/J.ENVPOL.2013.02.031) 23545014

[6] Oßmann BE, Sarau G, Holtmannspötter H, Pischetsrieder M, Christiansen SH, Dicke W. 2018 Small-sized microplastics and pigmented particles in bottled mineral water. Water Res. **141**, 307-316. (10.1016/J.WATRES.2018.05.027) 29803096

[7] Dick Vethaak A, Legler J. 2021 Microplastics and human health. Science (1979) **371**, 672-674. (10.1126/SCIENCE.ABE5041) 33574197

[8] Cox KD, Covernton GA, Davies HL, Dower JF, Juanes F, Dudas SE. 2019 Human Consumption of Microplastics. Environ. Sci. Technol. **53**, 7068-7074. (10.1021/ACS.EST.9B01517/ASSET/IMAGES/LARGE/ES-2019-015177_0004.JPEG) 31184127

[9] Schymanski D, Goldbeck C, Humpf HU, Fürst P. 2018 Analysis of microplastics in water by micro-Raman spectroscopy: Release of plastic particles from different packaging into mineral water. Water Res. **129**, 154-162. (10.1016/J.WATRES.2017.11.011) 29145085

[10] Isobe A, Iwasaki S, Uchida K, Tokai T. 2019 Abundance of non-conservative microplastics in the upper ocean from 1957 to 2066. Nat. Commun. **10**, 1-13. (10.1038/s41467-019-08316-9) 30679437 PMC6345988

[11] Peiponen KE, Räty J, Ishaq U, Pélisset S, Ali R. 2019 Outlook on optical identification of micro- and nanoplastics in aquatic environments. Chemosphere **214**, 424-429. (10.1016/J.CHEMOSPHERE.2018.09.111) 30273875

[12] Costa MF, Ivar Do Sul JA, Silva-Cavalcanti JS, Araújo MCB, Spengler Â, Tourinho PS. 2010 On the importance of size of plastic fragments and pellets on the strandline: A snapshot of a Brazilian beach. Environ. Monit. Assess. **168**, 299-304. (10.1007/S10661-009-1113-4/METRICS) 19680758

[13] Napper IE, Bakir A, Rowland SJ, Thompson RC. 2015 Characterisation, quantity and sorptive properties of microplastics extracted from cosmetics. Mar. Pollut. Bull. **99**, 178-185. (10.1016/J.MARPOLBUL.2015.07.029) 26234612

[14] Cheung PK, Fok L. 2016 Evidence of microbeads from personal care product contaminating the sea. Mar. Pollut. Bull. **109**, 582-585. (10.1016/J.MARPOLBUL.2016.05.046) 27237038

[15] Su Y et al. 2022 Author Correction: Steam disinfection releases micro(nano)plastics from silicone-rubber baby teats as examined by optical photothermal infrared microspectroscopy. Nat. Nanotechnol. **17**, 672-672. (10.1038/s41565-022-01155-8) 35641784

[16] Zhang Q et al. 2020 A Review of Microplastics in Table Salt, Drinking Water, and Air: Direct Human Exposure. Environ. Sci. Technol. **54**, 3740-3751. (10.1021/ACS.EST.9B04535/SUPPL_FILE/ES9B04535_SI_001.PDF) 32119774

[17] Hernandez LM, Xu EG, Larsson HCE, Tahara R, Maisuria VB, Tufenkji N. 2019 Plastic Teabags Release Billions of Microparticles and Nanoparticles into Tea. Environ. Sci. Technol. **53**, 12 300-12 310. (10.1021/ACS.EST.9B02540/ASSET/IMAGES/LARGE/ES9B02540_0003.JPEG) 31552738

[18] Li D et al. 2020 Microplastic release from the degradation of polypropylene feeding bottles during infant formula preparation. Nature Food **1**, 746-754. (10.1038/s43016-020-00171-y) 37128027

[19] Fromme H et al. 2019 Siloxane in baking moulds, emission to indoor air and migration to food during baking with an electric oven. Environ. Int. **126**, 145-152. (10.1016/J.ENVINT.2019.01.081) 30798195

[20] Liebezeit G, Liebezeit E. 2013 Non-pollen particulates in honey and sugar. Food Additives & Contaminants: Part A **30**, 2136-2140. (10.1080/19440049.2013.843025) 24160778

[21] Liebezeit G, Liebezeit E. 2014 Synthetic particles as contaminants in German beers. Food Additives & Contaminants: Part A **31**, 1574-1578. (10.1080/19440049.2014.945099) 25056358

[22] Yang D, Shi H, Li L, Li J, Jabeen K, Kolandhasamy P. 2015 Microplastic Pollution in Table Salts from China. Environ. Sci. Technol. **49**, 13622-13 627. (10.1021/ACS.EST.5B03163/ASSET/IMAGES/LARGE/ES-2015-03163A_0004.JPEG) 26486565

[23] Karami A, Golieskardi A, Keong Choo C, Larat V, Galloway TS, Salamatinia B. 2017 The presence of microplastics in commercial salts from different countries. Sci. Rep. **7**, 1-11. (10.1038/srep46173) 28383020 PMC5382780

[24] Iri AH et al. 2021 Optical detection of microplastics in water. Environmental Science and Pollution Research **28**, 63 860-63 866. (10.1007/S11356-021-12358-2/TABLES/1) 33462694

[25] Wang X, Patel A, Shashurin A. 2021 Combined microwave and laser Rayleigh scattering diagnostics for pin-to-pin nanosecond discharges. J. Appl. Phys. **129**, 183302. (10.1063/5.0054202/13401698/183302_1_ACCEPTED_MANUSCRIPT.PDF)

[26] Kerker M. 2013 The scattering of light and other electromagnetic radiation: physical chemistry: A series of monographs. New York, NY: Elsevier Science.

[27] Bohren CF, Huffman DR. 1983 Absorption and scattering of light by small particles. New York, NY: Wiley.

[28] van de Hulst HC. 1981 Light scattering by small particles. New York, NY: Dover Publications.

[29] Hahn DW. 2009. Light Scattering Theory.

[30] Frisvad JR, Christensen NJ, Jensen HW. 2012 Predicting the Apperance of Materials Using Lorenz-Mie Theory. In The Mie theory (eds W. Hergert, T. Wriedt), Springer Series in Optical Sciences, pp. 169. Berlin, Heidelberg: Berlin, Germany: Springer Berlin Heidelberg.

[31] Koelmans AA, Mohamed Nor NH, Hermsen E, Kooi M, Mintenig SM, De France J. 2019 Microplastics in freshwaters and drinking water: Critical review and assessment of data quality. Water Res. **155**, 410-422. (10.1016/J.WATRES.2019.02.054) 30861380 PMC6449537

[32] Yong CQY, Valiyaveettil S, Tang BL. 2020 Toxicity of Microplastics and Nanoplastics in Mammalian Systems. Int. J. Environ. Res. Public Health **17**, 1509. (10.3390/IJERPH17051509) 32111046 PMC7084551

[33] Rahman A, Sarkar A, Yadav OP, Achari G, Slobodnik J. 2021 Potential human health risks due to environmental exposure to nano- and microplastics and knowledge gaps: A scoping review. Sci. Total Environ. **757**, 143872. (10.1016/J.SCITOTENV.2020.143872) 33310568

[34] Niskanen I et al. 2019 Determination of nanoparticle size using Rayleigh approximation and Mie theory. Chem. Eng. Sci. **201**, 222-229. (10.1016/J.CES.2019.02.020)

[35] Nieto-Vesperinas M. 2020 Fundamentals of Mie scattering. In Dielectric metamaterials: fundamentals, designs, and applications, pp. 39-72. Woodhead Publishing.

[36] Liu Z, Li L, Li H, Mei L. 2019 Preliminary Studies on Atmospheric Monitoring by Employing a Portable Unmanned Mie-Scattering Scheimpflug Lidar System. Remote Sens. **11**, 837. (10.3390/RS11070837)

[37] Pedrotti FL, Pedrotti LM, Pedrotti LS. 2017 Introduction to optics. New York, NY: Cambridge University Press.

[38] Hecht Eugene. 2016 Optics, Global Edition. Physics / Astronomy - Advanced Physics: Optics. Malaysia: Pearson.

[39] Cavalieri F et al. 2008 Novel PVA-based hydrogel microparticles for doxorubicin delivery. Biomacromolecules **9**, 1967-1973. (10.1021/BM800225V/ASSET/IMAGES/LARGE/BM-2008-00225V_0010.JPEG) 18533701

[40] Saralidze K, Knetsch MLW, van Hooy-Corstjens CSJ, Koole LH. 2006 Radio-opaque and surface-functionalized polymer microparticles: Potentially safer biomaterials for different injection therapies. Biomacromolecules **7**, 2991-2996. (10.1021/BM0603903/ASSET/IMAGES/LARGE/BM0603903F00007.JPEG) 17096523

[41] Kattawar GW, Plass GN. 1967 Resonance Scattering from Absorbing Spheres. Appl. Opt. **6**, 1549-1554. (10.1364/AO.6.001549) 20062257

[42] Cachorro VE, Salcedo LL. 2012 New Improvements for Mie Scattering Calculations. **5**, 913-926. (10.1163/156939391X00950)

[43] Menguc MP, Mackowski DW, Altenkirch RA. 1990 Internal absorption cross sections in a stratified sphere. Appl. Opt. **29**, 1551-1559. (10.1364/AO.29.001551) 20563039

[44] Wiscombe WJ. 1980 Improved Mie scattering algorithms. Appl. Opt. **19**, 1505-1509. (10.1364/AO.19.001505) 20221065

[45] Dave JV. 2010 Scattering of Electromagnetic Radiation by a Large, Absorbing Sphere. IBM J Res Dev **13**, 302-313. (10.1147/RD.133.0302)

[46] ‘sinanngenc/miemtlb: mie scat calculation by MATLAB’. 2023 https://github.com/sinanngenc/miemtlb (accessed May 26, 2023).

[47] Balogun B, Timothy A. 2020 Production and characterization of melamine-formaldehyde moulding powder. International Journal of Advanced Academic Research | Sciences **6**, 2488-9849.

[48] Haynes William M. 2016. CRC handbook of chemistry and physics. Boca Raton, FL: CRC Press. (10.1201/9781315380476)

[49] Melamine. 2019. https://www.chembk.com/en/chem/Melamine (accessed Apr. 29, 2023).

[50] Lockwood DJ. 2016 Rayleigh and Mie Scattering. In: Encyclopedia of Color Science and Technology (ed. R Luo). Berlin Heidelberg: Springer.

[51] Genc S, Icoz K, Erdem T. 2023 Code for: Numerical analysis and experimental verification of optical scattering from microplastics. Zenodo. (10.5281/zenodo.8134110) PMC10410208 37564069

[52] Genc S, Icoz K, Erdem T. 2023 Numerical analysis and experimental verification of optical scattering from microplastics. Figshare. (10.6084/m9.figshare.c.6760141) PMC10410208 37564069

